# A versatile strategy towards non-covalent functionalization of graphene by surface-confined supramolecular self-assembly of Janus tectons

**DOI:** 10.3762/bjnano.6.64

**Published:** 2015-03-03

**Authors:** Ping Du, David Bléger, Fabrice Charra, Vincent Bouchiat, David Kreher, Fabrice Mathevet, André-Jean Attias

**Affiliations:** 1 Institut Parisien de Chimie Moléculaire, Chimie des Polymères, UMR CNRS 8232, Université Pierre et Marie Curie, 3 rue Galilée, 94200 Ivry, France; 2 Laboratoire de Nanophotonique, Service de Physique de l’Etat Condensé CEA/Saclay 91191 Gif sur Yvette Cedex, France; 3 Department Nanosciences Institut Neel, CNRS, Univ. Grenoble-Alpes, 38042 Grenoble Cedex 09, France

**Keywords:** C(sp^2^)-based substrates, graphene, Janus tecton, liquid–solid interface, scanning tunnelling microscopy, supramolecular self-assembly

## Abstract

Two-dimensional (2D), supramolecular self-assembly at surfaces is now well-mastered with several existing examples. However, one remaining challenge to enable future applications in nanoscience is to provide potential functionalities to the physisorbed adlayer. This work reviews a recently developed strategy that addresses this key issue by taking advantage of a new concept, Janus tecton materials. This is a versatile, molecular platform based on the design of three-dimensional (3D) building blocks consisting of two faces linked by a cyclophane-type pillar. One face is designed to steer 2D self-assembly onto C(sp^2^)-carbon-based flat surfaces, the other allowing for the desired functionality above the substrate with a well-controlled lateral order. In this way, it is possible to simultaneously obtain a regular, non-covalent paving as well as supramolecular functionalization of graphene, thus opening interesting perspectives for nanoscience applications.

## Review

### Introduction

Graphene is of significant interest for next generation electronics [1] particularly due to its electronic properties [2–3]. Thus, many research programs have been focused on the development of numerous approaches for synthesizing/transferring graphene onto surfaces during the last decade [4]. The next step towards device integration requires improved modification and functionalization of the bare graphene sheet [5].

This can be achieved either by covalent or non-covalent approaches [6]. In the former strategy, the covalent chemistry of pristine graphene requires chemical modification and the transformation of sp^2^ hybridized carbon atoms into sp^3^ hybridized. As a consequence, this disruption of the C-sp^2^ leads to the alteration of the characteristic electronic properties of graphene. For this reason, the non-covalent functionalization of graphene is expected to be more interesting, offering the opportunity to attach any functionality while simultaneously maintaining the integrity of the sp^2^-hybridized carbon network (i.e., not disturbing its electronic substrate properties) [6]. This aspect is critical as far as electronic devices are concerned. It is known that even low-density sp^3^ grafting strongly affects the delocalization of electrons within the graphene layer, making it incompatible for applications such as sensors [7]. Finally, an adsorbed molecular lattice can be applied to impose a super-period in the graphene atomic lattice. This new method allows the band and sub-band structure to be finely tuned for innovative two-dimensional (2D) semiconductor junctions [8].

However, the controlled positioning and organization of functional molecules into self-assembled monolayers at surfaces represent a major challenge for potential applications in various fields of nanotechnology [9–10]. Among the various manufacturing routes, bottom-up approaches [11] are particularly promising. They exploit supramolecular chemistry on surfaces to generate specific 2D structures and patterns at the nanometer scale through the self-assembly of building blocks, also called tectons [12]. These tectons are mainly planar π-conjugated molecules as they tend to bond to substrates in a flat-laying geometry. This allows the tectons to approach each other more easily and to engage in non-covalent interactions such as hydrogen bonding [13–15], metal–ligand coordination bonding [16–17] or even van der Waals interactions [18–19]. Thus, surface-confined supramolecular chemistry on surfaces appears to be the method of choice for the simple production of ordered arrays of molecules for the realization of complex functional surfaces. In other words, the exploration of both non-covalent and functionalized molecular self-assemblies on graphene, although a newly emerging approach, is a very promising strategy [20–24]. Moreover, the same principles reported for molecular in-plane-confined self-assembly on substrates (such as HOPG) can be directly transferred to graphene substrates, as was recently demonstrated for a few molecules. There are several examples regarding the formation of well-ordered 2D molecular adlayers self-assembled via hydrogen bonding [21] or other weak interactions on graphene [20], where most of these works were performed by evaporating small molecules onto graphene under ultra-high vacuum (UHV) conditions.

In this context, we recently developed a successful new strategy taking place at the liquid–solid interface at room temperature (RT) for the precise nanometer-scale 2D decoration of flat sp^2^-hybridized carbon supports (such as HOPG and graphene) with periodic arrays of functional 3D building blocks, known as Janus tectons [25]. Here, we summarize this general, versatile, and convenient approach for simultaneously (i) generating surface-based, supramolecular, periodic architectures on C(sp^2^)-based substrates, and (ii) independently exposing off-plane functionalities with controlled lateral order on demand.

### Mastering the surface-confined self-assembly of 2D tectons on C(sp^2^)-based substrates

**Figure 1:**
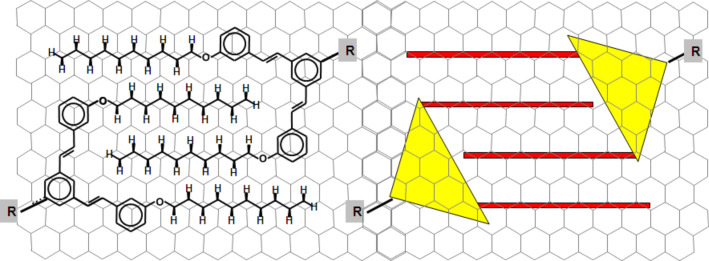
Molecular structure and schematic representation of the “molecular clip” illustrating its specific molecular bonding onto HOPG and showing the rigorous preservation of the Groszek structure [27] for the *n-*alkyl chains. Figure adapted with permission from [26], copyright 2007 Wiley-VCH Verlag GmbH & Co.

In the first stage, a strategy to obtain “on demand”, non-covalent self-assemblies with predetermined 2D periodic topologies on C(sp^2^)-based substrates was proposed [26]. Indeed, the construction of predictable and well-defined assemblies remains difficult to achieve, where the resulting topologies are often explained a posteriori based on molecule symmetry, molecule–substrate interactions and molecule–molecule interactions [19]. As a consequence, the “molecular clip concept” was introduced as a tool for surface specific supramolecular bonding on C(sp^2^)-based substrates and allowed for the first realization of a predetermined. “on demand” series of 0D, 1D or 2D topologies, based on a single rigid molecular core on HOPG. These achievements are based on the rational design of a novel functional molecular group, which turns into a non-covalent clip-like bond activated by graphite (Figure 1).

**Figure 2:**
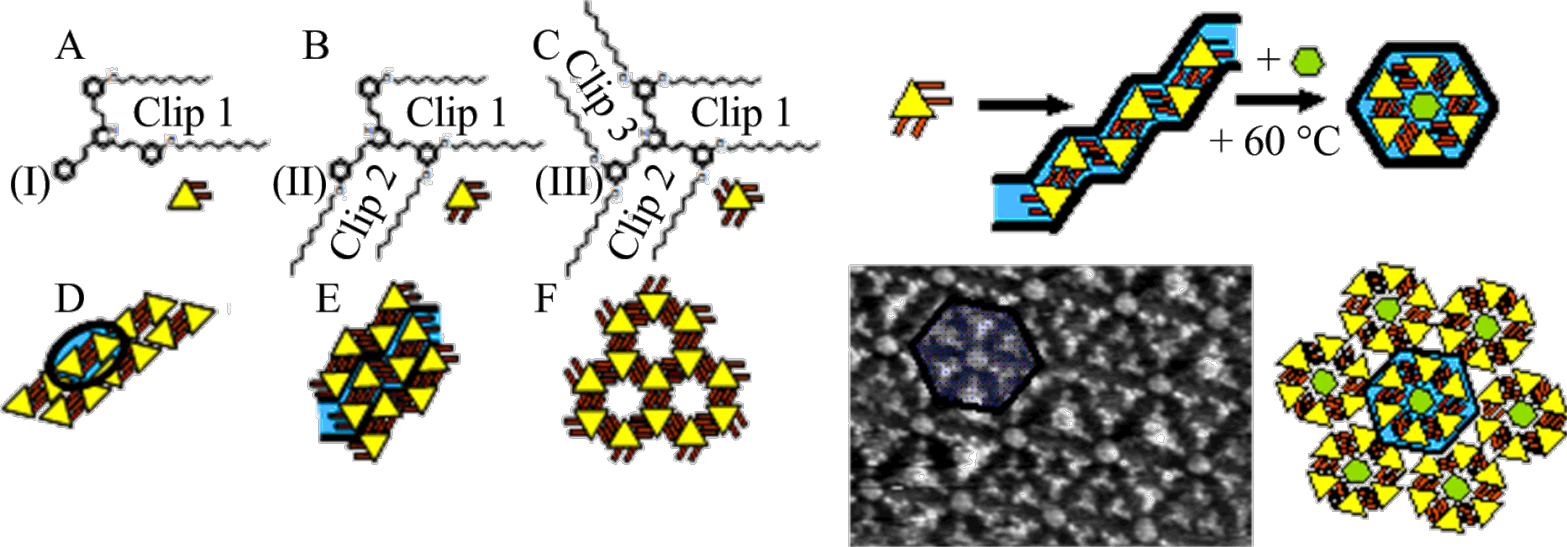
“On demand” realization of dimer-, polymer- or network-like topologies from a given rigid core and clips placed at different locations. Molecular structures of molecules I, II, and III (A–C), along with the anticipated self-assembly (D–F). Figure adapted with permission from [26], copyright 2007 Wiley-VCH Verlag GmbH & Co.

Among the interactions available for controlling supramolecular chemistry on surfaces, the interdigitation of alkyl chains was chosen because graphite surfaces such as HOPG exhibit a high affinity for *n*-alkane chains which form close-packed 2D lamellae described by the Groszek model [27]. This is due to the close match between the intra- and inter-chain distances and the graphite lattice parameters. More precisely, a new functional group, also called a “molecular clip”, was designed in order to mimic the adsorption of *n*-alkane chains on HOPG. This molecular unit presents two alkyl chains linked by a π-conjugated bridge. Since the distance between the two alkyl chains is twice the interchain distance in a well-organized *n*-alkane lamella, this unit acts as a supramolecular, functional linking group able to form strong, surface-assisted, intermolecular “clips” by interdigitation of the alkyl chains of two functional groups leading to the close-packing structure. Then, with this tool in hand, a fully deterministic strategy was developed where mono-, bi- and tri-multibranched functional building blocks (I–III) (based on a tristilbene rigid core bearing 1,2, and 3 peripheral molecular clips) have been designed, synthesized, and self-assembled on HOPG (Figure 2).

**Figure 3:**
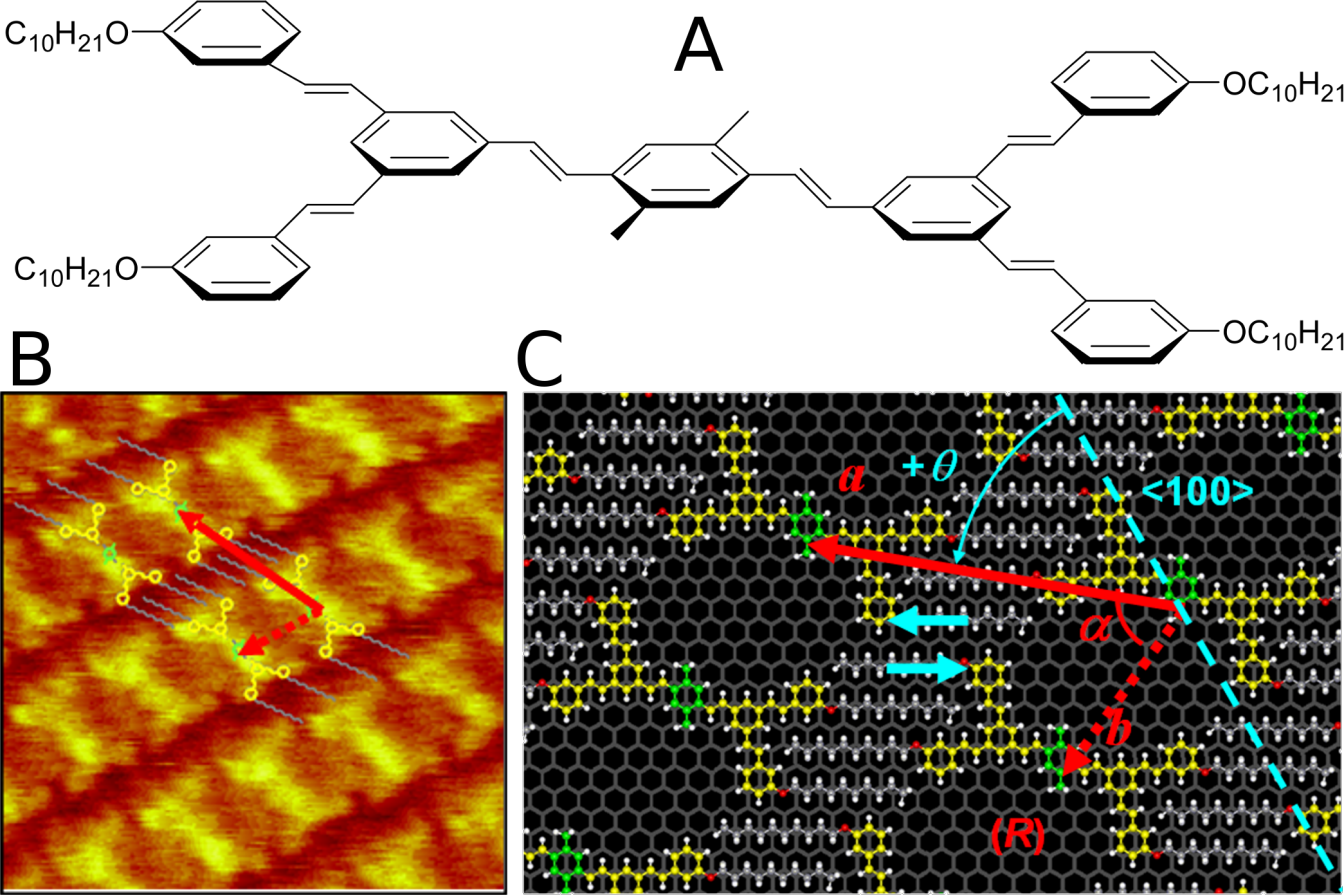
Compound IV: (A) molecular structure and (B) self-assembly of IV demonstrated by a high-resolution STM image of a monolayer domain of (IV) formed at the interface between graphite HOPG and a highly diluted (≈10^−4^ mol∙L^−1^) solution in phenyloctane. The sample bias was ≈−1.55 V and the tunnel current was ≈55 pA. The scan size and *z*-scale were ≈12.5 nm and ≈2.0 Å, respectively. The to-scale model of the molecular assembly is superimposed. (C) Molecular scheme of one unit cell of the monolayer adsorbed on HOPG (grey honeycomb background) of IV. Grey: alkyl chains; Yellow: conjugated cores; Green: multiple cyclophane levels. The unit cell is represented using red arrows: the solid arrow represents the intrachain period and the dotted arrow represents an interchain period. The blue line represents one <100> axis of HOPG. Figure adapted with permission from [28], copyright 2008 Wiley-VCH Verlag GmbH & Co.

The surface-confined molecular self-assemblies were characterized by scanning tunneling microscopy (STM) at the liquid–solid interface. As expected, they form non-covalent, surface self-assembled dimers, supramolecular linear polymers, and 2D networks. The versatility of the design was then demonstrated by synthesizing bifunctional molecules bearing two functional “clips” that end-cap a central moiety consisting of, for example, a benzene ring (IV) (Figure 3a). As shown in Figure 3b, compound IV also gives stable monolayers on HOPG. In addition, the self-assembly yields large, highly ordered domains, for which the lattice parameters can be accurately measured (Figure 3c), resulting in average lattice parameter values of *a* = 3.86 ± 0.15 nm, *b* = 2.11 ± 0.08 nm, and α = 65 ± 1°.

These results demonstrate that we are now able to control the supramolecular self-assembly on HOPG. First, a new tool acting as a functional moiety for surface-specific supramolecular bonding has been designed by combining and controlling molecule–substrate epitaxial adsorption and intermolecular packing interactions. Second, the “molecular clip” concept validity was demonstrated through the good match between the various expected and experimental topologies resulting from the supramolecular self-assembly at the liquid–HOPG interface of designed building blocks.

### 3D tectons for the controlled placement of functional molecules on C(sp^2^)-based substrates

**Figure 4:**
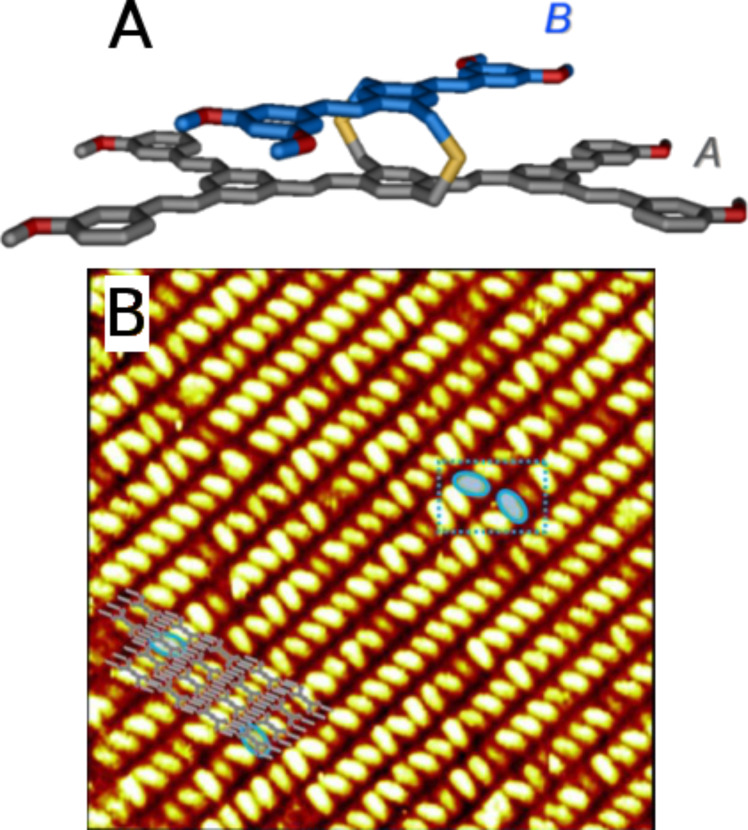
3D Janus tecton: schematic structure of the two-faced building block laying on the substrate (alkyl chains are omitted for clarity), and large-scale STM image (49.3 x 49.3 nm^2^) of the self-assembly at the HOPG–phenyloctane interface. The scaled model of the molecular assembly is superimposed on the STM picture (only lower levels A are represented for clarity). Figure adapted with permission from [29], copyright 2011 Wiley-VCH Verlag GmbH & Co.

In the second stage, the design of 3D building blocks was pursued [29]. This strategy is motivated by the need for functional surfaces for demanding forthcoming applications in nanotechnology. To address this issue, the realization of controlled functional molecular assemblies under the surfaces is a key point. To achieve such an objective requires the creation of out-of-plane functions and the full exploitation of the area above the substrate, in order to obtain an exact placement of functional objects in the third dimension above (perpendicular to) the surface. Most molecular recognition processes at surfaces require 3D receptors, and accessing the third dimension is also a mandatory step for nano-optics/electronics. Indeed the close proximity between the active conjugated system and a conducting substrate results in the rapid quenching of any electronic excitations. Thus, it is of prime importance to provide a strategy to decouple active molecular units from conducting C(sp^2^)-based substrates. In this context, we proposed for the first time a novel and highly versatile concept, the Janus-like 3D tecton concept. This building block consists of two different faces (A and B, like in all the Janus species) and a spacer linking them. Face A was designed to act as a pedestal capable of steering a 2D self-assembly onto the substrate, while B is a functional entity (e.g., a chromophore). The spacer acts as a pillar ensuring the decoupling of the B face from the substrate. Moreover, if the Janus tecton is laying on the substrate via the A face, the formation of a well-organized, in-plane monolayer covering the surface is expected as well as the steered positioning of the B face out of the plane. This concept was validated by designing and synthesizing the 3D tecton reported in Figure 4a. The pillar is a 3.3 Å [3.3]dithiaparacyclophane unit. The lower deck of this two-story linker is end-capped with two molecular clips in order to form the pedestal (A face), while a functional molecule, namely a distyrylbenzene fluorophore (highlighted in blue), forms the upper level (B face). STM studies at the liquid–HOPG interface demonstrated that the 2D well-defined nanostructured platform made of face A on the surface allowed controlled organization of the chromophores (faces B), leading to a regular array of functional units raised from the substrate (Figure 4b).

With these last results, it was demonstrated that (i) the multi-story molecules stack perpendicular to the substrate paving HOPG with long-range ordering, and (ii) the “floor” does not disturb the self-assembly in supramolecular, linear polymeric chains, even at large scales. Thus, this approach appears to be a breakthrough given the ability to control the 3-axis positioning (*x*,*y*,*z*) of a chromophore above a substrate. Moreover, due to its substantial, inherent tunability, this strategy opens up a promising novel route toward functional molecular nanostructures and new perspectives towards active surfaces and interfaces on C(sp^2^)-based substrates.

### 3D tectons for non-covalent functionalization of graphene by supramolecular self-assembly

**Figure 5:**
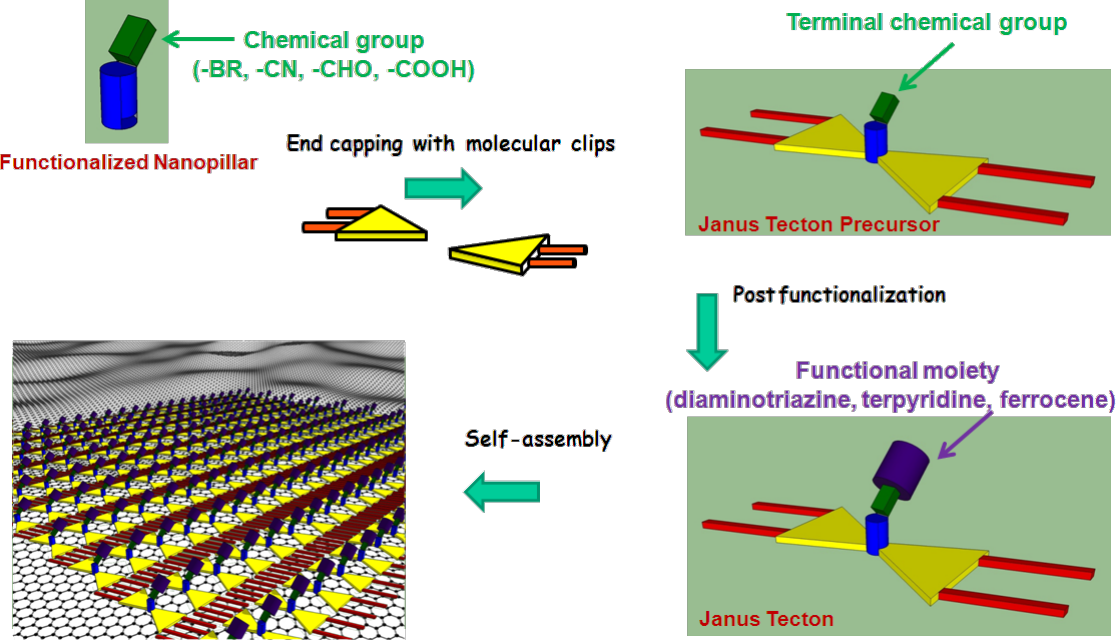
Synthetic strategy and expected organization on C(sp^2^)-carbon-based supports of the self-assembled Janus tectons, exposing a wide range of external interfacial compositions.

In the third stage, it was recently demonstrated that the Janus tecton concept is a versatile platform that can be used towards the non-covalent functionalization of graphene [25]. Before presenting the details of this strategy, it must be noted that the most commonly used non-covalent approach for graphene functionalization involves binding of pyrene-substituted species by π–π interaction [30–32], however, without formation of a well-ordered adlayer. Well-organized adlayers have only recently been obtained by transferring HOPG, molecular, in-plane confined, self-assembly studies to graphene substrates. However, to date, the majority of the investigations deal with only a few of molecules: 3,4,9,10-perylenetetracarboxylic dianhydride (PTCDA), phthalocyanine (and its metal coordination complexes), and C60 fullerenes [20]. Moreover, to our knowledge, no route towards 3D tecton surface-confined self-assembly, which adds functionality to graphene substrates, has been previously described and or even explored. In this context, we took advantage of the tremendous ability of the Janus tectons to form periodic, functional adlayers on HOPG, used as a versatile new tool for a similar non-covalent functionalization of graphene. To ensure the versatility compared to our previous work, the synthetic sequence as well as the pillar design were revisited and rationalized. In fact, we developed a synthetic convergent strategy (Figure 5) which consists of first synthesizing a series of intermediate 3D building blocks (Janus precursors, JAP) bearing small terminal chemical groups at the top of the pillar (a dithia[3.3]metaparacyclophane derivative).

In a first attempt to validate the strategy, the terminal chemical groups were –Br, –CN, –CHO, and –COOH. Second, after appropriate post-functionalization, the Janus precursors formed the target Janus tectons (JA), exposing as an upper face different functional moieties such as triazine-4,5-diamine, 2,6-bis(2-pyridyl)pyridine and ferrocene units.

**Figure 6:**
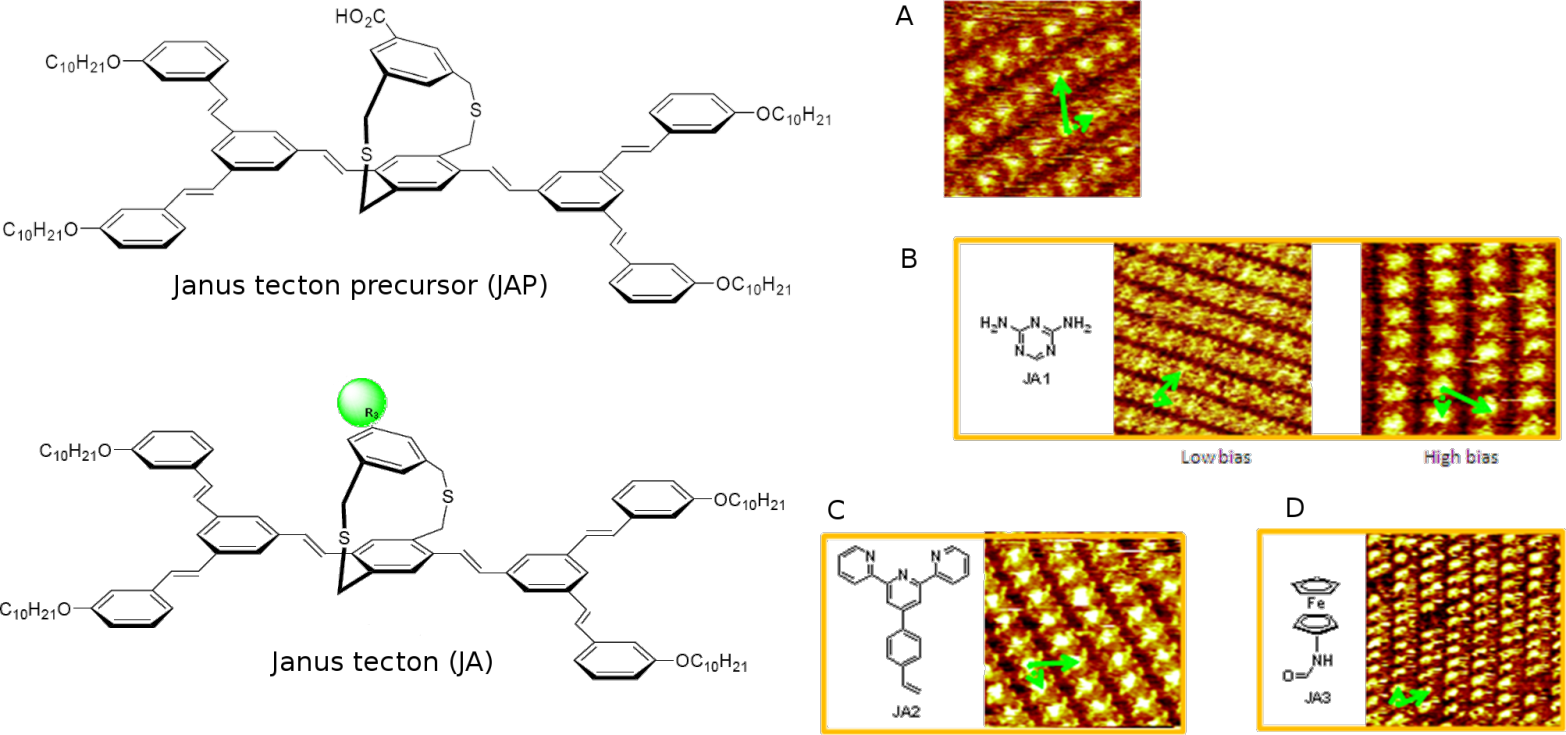
Self-assembly of a Janus tecton precursor (JAP) and the Janus tectons (JA). Drift-corrected STM images obtained at the interface between HOPG and a 10^−4^ M solution in phenyloctane of (A) JAP, 13 × 13 nm, set point *I*_T_ = 35 pA, sample bias *V*_B_ = −1200 mV, (B) JA functionalized with triazine-4,5-diamine, low bias: 22 × 22 nm, *I*_T_ = 8 pA, *V*_B_ = −950 mV and high bias: 15 × 15 nm, *I*_T_ = 14 pA, *V*_B_ = −1350 mV, (C) JA functionalized with terpyridine, 16 × 16 nm, *I*_T_ = 25 pA, *V*_B_ = −1500 mV, and (D) JA functionalized with ferrocene, 25 × 25 nm, *I*_T_ = 20 pA, *V*_B_ = −1330 mV. One of the unit cells corresponding to the lattice formed by the non-functionalized pedestal, *a* = 3.84 nm, *b* = 2.08 nm and α = 64°, is highlighted in each image (green arrows) to illustrate the agreement between all Janus tecton lattices. Figure adapted with permission from [25], copyright 2014 Wiley-VCH Verlag GmbH & Co.

The self-assembly properties of the JAPs and JAs were investigated by STM at the liquid–HOPG interface, at room temperature (Figure 6). First, it is obvious that all the probed Janus building blocks spontaneously self-assemble into 2D networks on HOPG. More surprisingly, they form periodic lattices with the same parameters within the typical experimental accuracy of ±5% for the distances and 2° for the angles (*a* = 3.84 nm, *b* = 2.08 nm and α = 64°) regardless of the building block. These values are compatible with those of the lattice formed by the neat ground floor [32]. Then, it was inferred that the same process drives the self-assembly on the substrate regardless of the tecton. An explanation is that the ground level of functionalized 3D Janus tectons of any shape, size or function in JAP and JA tectons, act to steer the 2D self-assembly. This is due to interactions with both the HOPG and with the neighboring adsorbed molecules, as confirmed by molecular mechanics calculations [25]. Both the experimental and theoretical lattice values of JA evidenced that the presence of relatively large entities on the upper level which did not perturb the self-assembly. In addition, they further confirmed that the self-assembly is stabilized by adsorption of alkyl chains in registry with HOPG and by their maximized close-packing interactions through interdigitation. The comparison of the cross-sectional area of the pedestal (*a*∙*b*∙sinα = 7.18 nm^2^) with the calculated cross-sectional areas occupied by each upper unit (to a maximum of 2.02 nm^2^ for the largest upper level, terpyridine unit) can partially explain these features. All of the upper units are size-compatible with the huge footprint value.

**Figure 7:**
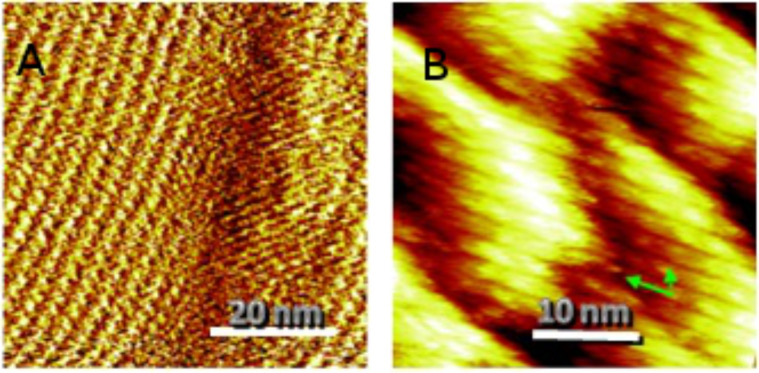
Self-assembly on graphene. Drift-corrected STM images obtained in air on a monolayer graphene substrate grown by chemical vapor deposition on a polycrystalline copper foil at the interface between this substrate and a 10^−4^ M solution of Janus tectons in phenyloctane. (A) 58 × 58 nm, set point *I*_T_ = 20 pA, sample bias *V*_B_ = −950 mV, (B) 34 × 34 nm, *I*_T_ = 13 pA, *V*_B_ = −950 mV. A unit cell corresponding to the lattice formed on HOPG (*a* = 3.84 nm, *b* = 2.08 nm, and α = 64°) is highlighted in (B) by green arrows. Figure adapted with permission from [25], copyright 2014 Wiley-VCH Verlag GmbH & Co.

Finally, the self-assembly of the Janus tectons onto a graphene monolayer, grown by chemical vapor deposition onto a polycrystalline foil, was investigated. As evidenced by a typical STM image (Figure 7) recorded at the liquid–graphene interface at room temperature, a self-assembled monolayer is observed. By using the same procedure as in the case of a HOPG substrate, the lattice parameters of the network have been estimated. The main result is that they are similar to those measured in the case of the HOPG substrate.

These results demonstrate that for the first time a general platform for the non-covalent functionalization of flat sp^2^-carbon-based substrates (including graphene) has been investigated. In contrast to other studies performed by evaporating low molecular weight molecules under UHV conditions, in our approach, the self-assembly is achieved at the liquid–solid interface, additionally allowing the physisorption of higher molecular weight molecules.

### Conclusion

Using the molecular clip concept as a tool for supramolecular bonding on C(sp^2^)-based substrates, the Janus tecton concept offers a versatile platform towards the non-covalent functionalization of graphene. The reported strategy is expected to be applicable for the generation of self-assembly systems exhibiting on demand functionalization, expanding the application possibilities of this functionalization method. Moreover, working at the liquid–solid interface makes this strategy easy to implement and should also provide the opportunity to control the self-assembly by tuning the molecule–solvent and solvent–substrate interactions. Finally, the successful self-assembly on graphene, together with the possibility to transfer the graphene monolayer onto various substrates, should open up new opportunities in nanoscience.
